# Treatment Beyond Progression After Anti-PD-1 Blockade in Hepatocellular Carcinoma

**DOI:** 10.1158/2767-9764.CRC-23-0025

**Published:** 2023-09-21

**Authors:** Mir Lim, Maishara Muquith, Bernadette Miramontes, Magdalena Espinoza, David Hsiehchen

**Affiliations:** 1 Division of Hematology and Oncology, Department of Internal Medicine, University of Texas Southwestern Medical Center, Dallas, Texas.; 2 Division of Digestive and Liver Diseases, Department of Internal Medicine, University of Texas Southwestern Medical Center, Dallas, Texas.; 3 Harold C. Simmons Comprehensive Cancer Center, University of Texas Southwestern Medical Center, Dallas, Texas.

## Abstract

Immune checkpoint inhibitors (ICI) can induce atypical tumor responses including pseudoprogression in a subset of patients who may benefit from treatment beyond progression. While ICIs have emerged as frontline treatments for hepatocellular carcinoma (HCC) and are associated with clinical benefit in a minority of patients, it is unclear whether treatment beyond progression has utility in this disease type. In a multicenter cohort analysis, treatment beyond progression was associated with no new safety signals, objective responses in 5.8% of patients, and disease control in 44% of patients. Progression-free survival and overall survival were comparable between patients treated beyond progression and patients treated with subsequent therapies, demonstrating that treatment beyond progression was not detrimental to survival outcomes. Rather, treatment beyond progression may benefit select patients with HCC and could represent a viable strategy for maximizing treatment benefit in these patients.

**Significance:**

Treatment beyond progression with ICIs in patients with HCC is safe and may benefit a subset of patients due to later-onset tumor responses or disease stability. These findings may guide the design of trials testing ICIs in HCC and the use of treatment beyond progression in routine practice.

## Introduction

Hepatocellular carcinoma (HCC) is the most prevalent primary liver cancer with a rising incidence in many countries and is a leading cause of cancer deaths worldwide (1). Immune checkpoint inhibitors (ICI) are currently the preferred frontline agents for the treatment of advanced HCC and are associated with prolonged overall survival (OS2) benefit and tumor responses compared with tyrosine kinase inhibitors (2). However, determining the efficacy of ICIs in clinical practice is hampered by evidence that ICIs can induce immune-related responses in tumor lesions that may not be accurately classified using standard criteria including RECIST 1.1 (3). RECIST 1.1 defines unequivocal disease progression as an increase in the dimensions of tumor lesions by at least 20% or the observation of new tumor lesions, and this is conventionally applied in clinical trial endpoints and guides treatment continuation in routine practice (3). Nonetheless, immune-related treatment responses include delayed antitumor effects due to time-dependent immune cell activation, and transient increases in tumor size or the emergence of new lesions caused by edema and immune cell infiltration (4). This suggests that some patients treated with ICIs who initially meet RECIST 1.1 for treatment failure may actually benefit from further treatment. This underlies the premise of treatment beyond progression for ICIs because the optimal duration of these treatments remains undefined. *Post hoc* analyses of trials in lung cancers, renal cell carcinomas, and melanoma have not provided conclusive evidence on the use of treatment beyond progression (5, 6). However, emerging data suggest that there may be a limited, but non-trivial clinical benefit in a subset of selected patients (6). Whether treatment beyond progression in HCC is associated with meaningful benefit is unknown. Herein, we performed a real-world cohort study across two institutions to examine the safety and outcomes of treatment beyond progression in patients with advanced HCC.

## Materials and Methods

### Study Population

After receiving Institutional Review Board (IRB) approval, patients with advanced HCC who were unresectable and received ICIs between January 1, 2018 and December 31, 2022 were identified from the electronic medical record at the University of Texas Southwestern Medical Center (Dallas, TX) and Parkland Health and Hospital System (Dallas, TX). All patients meeting these inclusion criteria were included in this study regardless of sex, race, or ethnicity. Patients receiving radiotherapy during their ICI course were excluded from this study. Treatment beyond progression was defined as the continuation of ICIs after the first occurrence of radiographic evidence (according to RECIST 1.1) of progression. Treatment beyond progression was used until there was evidence of clinical or radiographic progression compared with the time of first disease progression (according to RECIST 1.1) on subsequent restaging scans. This study was conducted in accordance with the Declaration of Helsinki.

### Data Collection

Patient characteristics, treatment history, and toxicities were manually abstracted from medical records including clinic notes, telephone encounters, radiology images and reports, laboratory results, medication lists, and hospitalization records. Radiographic responses were based on RECIST 1.1. Progression-free survival (PFS2) after initial progression to ICIs was calculated from the time of first progression to ICIs to subsequent progression of disease or death. OS2 was calculated from the time of first progression to ICIs to death.

### Statistical Analysis

The *χ*^2^ or two-sided Mann–Whitney *U* test was used to assess differences in the frequency of categorical characteristics between patient cohorts. Median survival times were calculated using the Kaplan–Meier method and the log-rank test was used to assess differences in survival. Data analysis was performed using SPSS 24 (IBM).

### Data Availability Statement

Data generated in this study are not publicly available based on the IRB-approved protocol to maintain patient confidentiality. Deidentified and aggregated data are available upon reasonable request from the corresponding author.

## Results

Among 144 patients with advanced HCC treated with anti-PD-1/L1 therapies, 102 patients had progressive disease at the data cut-off date according to RECIST 1.1, and 68 patients received further treatment (34 treated beyond progression and 34 treated with other subsequent therapies). Clinical characteristics were not significantly different between patients who received treatment beyond progression and patients who received subsequent line therapies (Table 1). In contrast, patients who received no other subsequent line therapy including treatment beyond progression were more frequently associated with a poor baseline Eastern Cooperative Oncology Group (ECOG) status (Table 1). Nearly half of the patients treated beyond progression were initially treated with nivolumab (Table 2). PFS to the first occurrence of progression after initial ICI treatment was shorter among patients treated beyond progression (2.7 months) and patients who did not receive subsequent treatment (2.3 months) compared with patients who received subsequent line therapies (4.5 months; log-rank test, *P* = 0.007). Deterioration of ECOG status at first ICI progression was present in 6 patients treated beyond progression (all of which increased from 0 to 1) and 11 patients treated with subsequent line therapy (9 of which increased from 0 to 1, and 2 of which increased from 0 or 1 to 2).

**TABLE 1:** Characteristics of patients with HCC treated beyond progression, treated with subsequent therapies, and received no subsequent therapies

		Treatment beyond progression	Treatment with subsequent therapy	No subsequent therapy	*P*	*P*
Patient characteristics		A	B	C	A vs. B	A vs. C
Age^a^		63 (59–69)	62 (56–68)	63 (60–69)	0.41	0.99
Sex^b^	Male	30 (88.2)	27 (79.4)	29 (85.3)	0.32	0.72
	Female	4 (11.8)	7 (20.6)	5 (14.7)	NA	NA
Cirrhosis etiology^b^	HBV	8 (23.5)	4 (11.8)	0 (0)	0.54	<0.01
	HCV	15 (44.1)	19 (55.9)	21 (61.8)	NA	NA
	NASH	5 (14.7)	7 (20.6)	5 (14.7)	NA	NA
	ALD	0 (0)	0 (0)	6 (17.6)	NA	NA
	Other	1 (2.9)	0 (0)	2 (5.9)	NA	NA
Child Pugh^b^	A	24 (70.6)	28 (82.4)	19 (55.9)	0.25	0.21
	B/C	10 (29.4)	6 (17.6)	15 (44.1)	NA	NA
Extrahepatic disease^b^	Yes	14 (41.2)	16 (47.1)	16 (47.1)	0.62	0.62
	No	20 (58.8)	18 (52.9)	18 (52.9)	NA	NA
ECOG^b^	0	22 (64.7)	27 (79.4)	12 (35.3)	0.29	0.05
	1	9 (26.5)	7 (20.6)	14 (41.2)	NA	NA
	2	3 (8.8)	0 (0)	3 (8.8)	NA	NA
	3	0 (0)	0 (0)	4 (11.7)	NA	NA

^a^Statistical significance tested using two-sided Mann–Whitney *U* test.

^b^Statistical significance tested using the *χ*^2^ test.

**TABLE 2:** First-line regimens used for patients with HCC treated beyond progression, treated with subsequent therapies, and received no subsequent therapies

	Treatment beyond progression	Treatment with subsequent therapy	No subsequent therapy
Nivolumab	16 (47.1)	7 (20)	18 (52.9)
Atezolizumab plus bevacizumab	9 (26.5)	18 (54.3)	11 (32.4)
Pembrolizumab	9 (26.5)	8 (22.9)	4 (11.8)
Durvalumab	0 (0)	1 (2.9)	1 (2.9)

**FIGURE 1:**
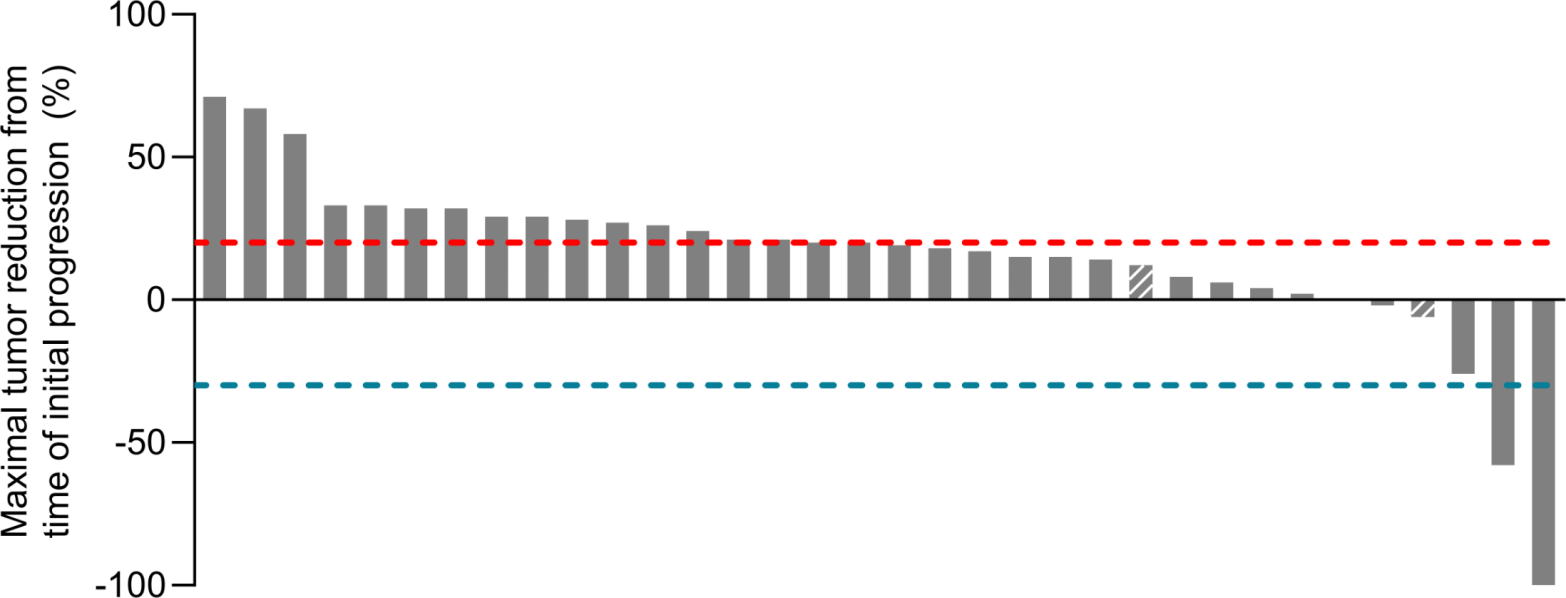
Tumor response after treatment beyond progression in HCC. Waterfall plot of the maximal tumor reduction after treatment beyond progression with ICIs. Dashed columns indicate patients with progressive disease due to the appearance of new lesions.

**FIGURE 2:**
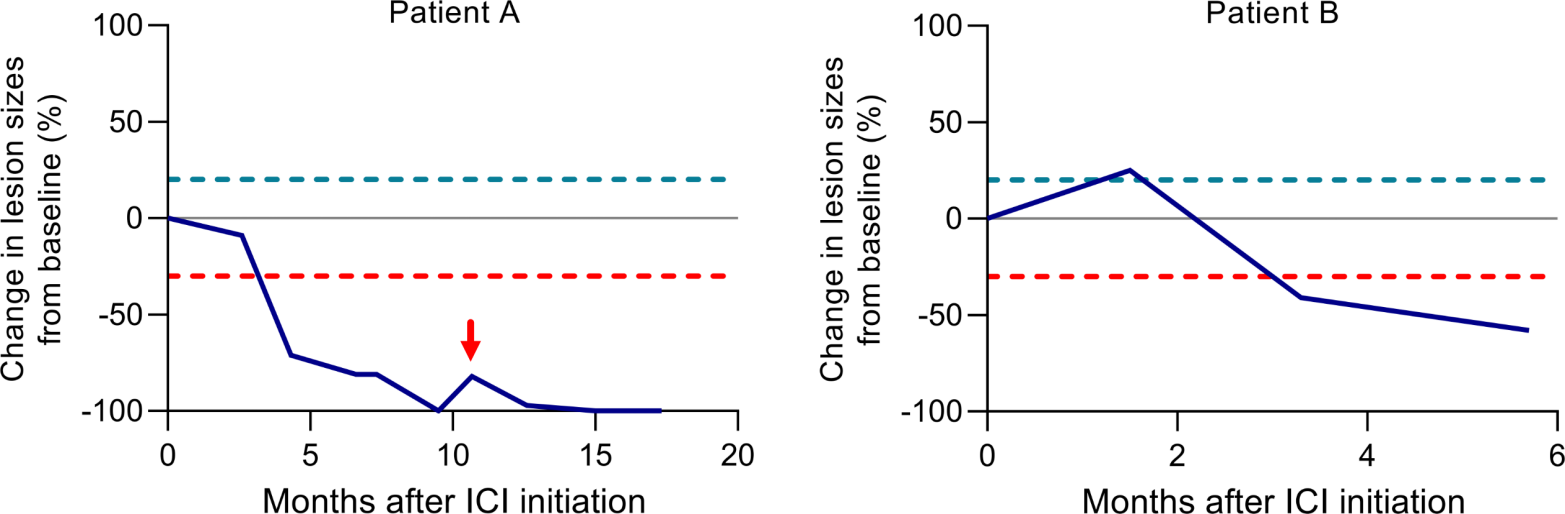
Changes in tumor measurements among patients with objective responses after treatment beyond progression. Spider plots indicate changes in tumor dimensions in 2 patients with progressive disease according to RECIST 1.1 followed by response after continuing ICI.

Among patients treated beyond progression, objective tumor responses according to RECIST 1.1 including partial and complete responses were observed in 5.8% (1 partial response, 1 complete response), while stable disease was observed in 38% (Fig. 1). A total of 11.8% of patients demonstrated evidence of tumor shrinkage after initial progression of disease (Fig. 1). Among the 2 patients with objective responses after treatment beyond progression, 1 patient developed new hepatic lesions 10 months after initiation of nivolumab, which subsequently regressed (Fig. 2). The other patient had a 25% growth of target lesions after starting atezolizumab plus bevacizumab, which diminished at the next restaging scan with further treatment of the same regimen (Fig. 2).

**FIGURE 3:**
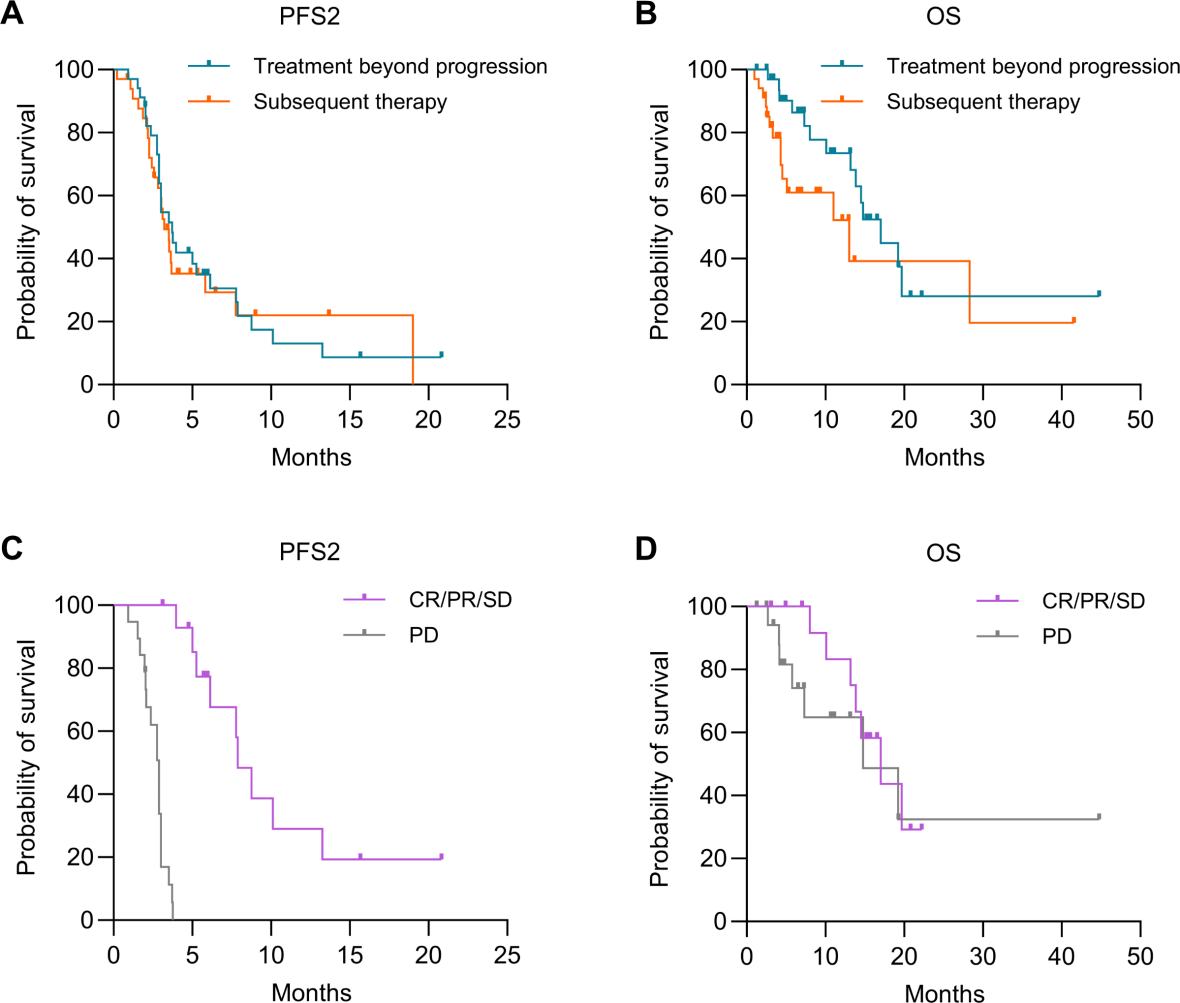
PFS2 and OS2 after treatment beyond progression in HCC. **A,** Kaplan–Meier curves depict PFS2 among patients treated beyond progression and treated with subsequent therapies. **B,** Kaplan–Meier curves depict OS2 among patients treated beyond progression and treated with subsequent therapies. **C,** Two-month landmark analysis of PFS2 among patients treated beyond progression who had evidence of disease control and patients with progressive disease. **D,** Two-month landmark analysis of OS2 among patients treated beyond progression who had evidence of disease control and patients with progressive disease.

To assess survival outcomes after initial progression, we calculated PFS2 and OS2 across patient groups where time to events was calculated from the time of initial progression. The median PFS2 of patients treated beyond progression and treated with subsequent line therapy was 3.7 and 3.2 months, respectively (log-rank test, *P* = 0.81; Fig. 3A). The median OS2 of patients treated beyond progression and treated with non-ICI subsequent line therapy was 17 and 13 months, respectively (log-rank test, *P* = 0.11; Fig. 3B). Two-month land-mark analyses showed that disease control defined as complete or partial responses and stable disease after treatment beyond progression was associated with prolonged PFS2 (7.9 vs. 3 months; log-rank test, *P* < 0.001) and OS2 (17 vs. 14.8 months; log-rank test, *P* = 0.45; Fig. 3C and D). This suggests that subsequent tumor responses in patients treated beyond progression may be predictive of clinical benefit in select patients.

Subset analyses of patients with viral or non-viral etiologies of cirrhosis and the presence and absence of extrahepatic disease were performed to assess whether clinical factors may be associated with benefit after treatment beyond progression. Given low objective response rates in patients treated beyond progression, we compared disease control rates (complete response/partial response/stable disease) between groups as well as median PFS2. There was no statistical difference in disease control rates between viral versus non-viral etiologies (36% vs. 43%, Fisher exact test *P* value = 0.53) and the presence versus absence of extrahepatic disease (47% vs. 33%, Fisher exact test *P* value = 0.52). Similarly, there was no statistical difference in the median PFS2 between viral versus non-viral etiologies (3.7 vs. 3 months, log-rank *P* value = 0.74) and the presence versus absence of extrahepatic disease (3.7 vs. 3.5 months, log-rank *P* value = 0.8).

Treatment adverse events occurred in 14.7% of patients during treatment beyond progression, all of which were grade 1 or 2. No patient permanently discontinued ICIs during treatment beyond progression due to toxicities.

## Discussion

These results indicate that treatment beyond progression with ICIs in HCC is safe and may be a therapeutic strategy in select individuals. Disease control in a substantial proportion of patients as well as objective tumor responses in a subset of patients confirms that delayed antitumor effects in HCC may be observed with ICIs. In addition, the lack of a difference in PFS2 and OS2 between patients treated beyond progression and patients treated with subsequent therapies suggests that continuation of ICIs after initial progression is not detrimental to survival. Most demographic and clinical characteristics were not significantly different between patients who were treated beyond progression and those who received subsequent line therapy in this study, but patients with a shorter time to first ICI progression and without deterioration of ECOG status were more likely to be treated beyond progression. Understanding provider and patient preferences that may influence the use of treatment beyond progression will need to be delineated to implement such treatment strategies.

Further research is necessary to explore the generalizability of these results because the utilization of treatment beyond progression may vary across institutions. Review of imaging was not blinded to clinical treatment decisions, which was a limitation of this study. Because of the retrospective nature of this study, it was difficult to capture specific reasons why treatment was continued beyond progression. Although the relatively small size of our cohort limits the generalizability of our findings and the ability to perform a matched cohort analysis, this study is among the first to evaluate outcomes of treatment beyond progression to anti-PD-(L)1 therapy in HCC. Larger-scale studies are warranted to provide insight into which subset of patients with HCC would benefit from treatment beyond progression and the appropriate duration of such treatment. Nonetheless, our results are aligned with findings from several studies evaluating the continuation of ICIs beyond progression in non–small cell lung cancer (NSCLC), renal cell carcinoma, and melanoma, where a majority of patients demonstrated stable disease without new safety signals (5–7). Meta-analyses in melanoma and NSCLC also indicate that treatment beyond progression may be associated with improved survival outcomes, but prospective evidence is lacking (5, 7). However, even in these other cancer types, it remains unclear whether treatment beyond progression is beneficial for unselected patients, and if not, whether clinical or biological factors may guide treatment selection. Further research identifying biomarkers that predict outcomes after treatment beyond progression in HCC will be needed to guide patient selection for the continuation of ICIs. In addition, whether modified RECIST proposed for other malignancies may lead to more accurate assessments of ICI benefit in HCC remains to be clarified because our results suggest that pseudoprogression with ICIs in HCC is rare (8–10).

In summary, treatment beyond progression with ICIs may benefit a subset of patients with HCC due to the potential for later-onset tumor responses or disease stability. Given that treatment beyond progression may delay the use of subsequent line therapies, including oral kinase inhibitors which are associated with a less tolerable toxicity profile, treatment beyond progression with ICIs represents a promising avenue for maximizing treatment benefit in patients with HCC (11).

## References

[1] Llovet JM , Kelley RK, Villanueva A, Singal AG, Pikarsky E, Roayaie S, . Hepatocellular carcinoma. Nat Rev Dis Primers 2021;7:6. 33479224 10.1038/s41572-020-00240-3 PMC13537433

[2] Llovet JM , Castet F, Heikenwalder M, Maini MK, Mazzaferro V, Pinato DJ, . Immunotherapies for hepatocellular carcinoma. Nat Rev Clin Oncol 2022;19:151–72. 34764464 10.1038/s41571-021-00573-2

[3] Chiou VL , Burotto M. Pseudoprogression and immune-related response in solid tumors. J Clin Oncol 2015;33:3541–3. 26261262 10.1200/JCO.2015.61.6870 PMC4622096

[4] Wolchok JD , Hoos A, O'Day S, Weber JS, Hamid O, Lebbe C, . Guidelines for the evaluation of immune therapy activity in solid tumors: immune-related response criteria. Clin Cancer Res 2009;15:7412–20. 19934295 10.1158/1078-0432.CCR-09-1624

[5] Beaver JA , Hazarika M, Mulkey F, Mushti S, Chen H, He K, . Patients with melanoma treated with an anti-PD-1 antibody beyond RECIST progression: a US Food and Drug Administration pooled analysis. Lancet Oncol 2018;19:229–39. 29361469 10.1016/S1470-2045(17)30846-X PMC5806609

[6] Spagnolo F , Boutros A, Cecchi F, Croce E, Tanda ET, Queirolo P. Treatment beyond progression with anti-PD-1/PD-L1 based regimens in advanced solid tumors: a systematic review. BMC Cancer 2021;21:425. 33865350 10.1186/s12885-021-08165-0 PMC8052683

[7] Kuo WK , Weng CF, Lien YJ. Treatment beyond progression in non-small cell lung cancer: a systematic review and meta-analysis. Front Oncol 2022;12:1023894. 36465371 10.3389/fonc.2022.1023894 PMC9713814

[8] Katz SI , Hammer M, Bagley SJ, Aggarwal C, Bauml JM, Thompson JC, . Radiologic pseudoprogression during anti-PD-1 therapy for advanced non-small cell lung cancer. J Thorac Oncol 2018;13:978–86. 29738824 10.1016/j.jtho.2018.04.010

[9] Seymour L , Bogaerts J, Perrone A, Ford R, Schwartz LH, Mandrekar S, . iRECIST: guidelines for response criteria for use in trials testing immunotherapeutics. Lancet Oncol 2017;18:e143–52. 28271869 10.1016/S1470-2045(17)30074-8 PMC5648544

[10] Lim M , Muquith M, Miramontes B, Lee CJ, Espinoza M, Huang YH, . Surrogate and modified endpoints for immunotherapy in advanced hepatocellular carcinoma. Hepatology 2023 [Online ahead of print]. 10.1097/HEP.0000000000000494 37254559

[11] Griffiths CD , Zhang B, Tywonek K, Meyers BM, Serrano PE. Toxicity profiles of systemic therapies for advanced hepatocellular carcinoma: a systematic review and meta-analysis. JAMA Netw Open 2022;5:e2222721. 35849393 10.1001/jamanetworkopen.2022.22721 PMC9295000

